# Improving Pulse Rate Measurements during Random Motion Using a Wearable Multichannel Reflectance Photoplethysmograph

**DOI:** 10.3390/s16030342

**Published:** 2016-03-07

**Authors:** Kristen M. Warren, Joshua R. Harvey, Ki H. Chon, Yitzhak Mendelson

**Affiliations:** 1 Department of Biomedical Engineering, Worcester Polytechnic Institute, Worcester, MA 01605, USA; kmwarren@wpi.edu (K.M.W.); jrharvey@wpi.edu (J.R.H.); 2 Department of Biomedical Engineering, University of Connecticut, Storrs, CT 06269, USA; kchon@engr.uconn.edu

**Keywords:** motion artifacts, multichannel photoplethysmograph, multichannel template matching, pulse rate, wearable sensor, pulse oximeter

## Abstract

Photoplethysmographic (PPG) waveforms are used to acquire pulse rate (PR) measurements from pulsatile arterial blood volume. PPG waveforms are highly susceptible to motion artifacts (MA), limiting the implementation of PR measurements in mobile physiological monitoring devices. Previous studies have shown that multichannel photoplethysmograms can successfully acquire diverse signal information during simple, repetitive motion, leading to differences in motion tolerance across channels. In this paper, we investigate the performance of a custom-built multichannel forehead-mounted photoplethysmographic sensor under a variety of intense motion artifacts. We introduce an advanced multichannel template-matching algorithm that chooses the channel with the least motion artifact to calculate PR for each time instant. We show that for a wide variety of random motion, channels respond differently to motion artifacts, and the multichannel estimate outperforms single-channel estimates in terms of motion tolerance, signal quality, and PR errors. We have acquired 31 data sets consisting of PPG waveforms corrupted by random motion and show that the accuracy of PR measurements achieved was increased by up to 2.7 bpm when the multichannel-switching algorithm was compared to individual channels. The percentage of PR measurements with error ≤ 5 bpm during motion increased by 18.9% when the multichannel switching algorithm was compared to the mean PR from all channels. Moreover, our algorithm enables automatic selection of the best signal fidelity channel at each time point among the multichannel PPG data.

## 1. Introduction

Pulse oximetry uses light absorption to measure arterial blood oxygen saturation (SpO_2_) and pulse rate (PR) from photoplethysmographic (PPG) signals. Pulse oximeters detect a pulsatile signal that is normally only a small percentage of the total PPG signal. Therefore, any transient motion of the sensor relative to the skin, such as during exercise, can cause a significant artifact in the optical measurement. Furthermore, if these artifacts mimic a heartbeat, the instrument may not be able to differentiate between the pulsations that are due to motion artifacts (MA) and normal arterial pulsations, thereby distorting the PPG waveforms and causing false or erroneous PR readings. The primary cause of MA in pulse oximetry is predominantly due to changes in the light path during sensor movements [1].

Pulse oximetry is widely used in hospitals where motion artifacts are generally less pronounced compared to mobile health applications. For instance, during patient transport, bouncing of the vehicle can cause the probe to be displaced and temporarily lose the PPG signal. Thus, the stability of the PPG signal is essential to facilitate reliable vital sign monitoring.

Motion artifacts are difficult to filter out since they do not have a predetermined frequency range and their spectral content often overlaps with the frequency band of the PPG waveform. If motion artifact persists long enough and has a frequency in the range of normal PR, the calculated PR can be highly inaccurate. Clinicians have cited motion artifacts in pulse oximetry as the most common cause of false alarms, loss of signal, and inaccurate readings [2].

The primary approach to reduce the effects of motion artifact is the implementation of software-based algorithms that attempt to extract a clean PPG waveform from the motion-corrupted PPG signal [3,4,5,6,7,8,9,10,11,12,13,14,15,16,17,18]. Conventional signal filtering is helpful, but not very effective when MA has no predetermined frequency band. Particularly, when the noise is near or shares the same frequency band as the signal components of interest, this technique can suppress the desired signals. Baseline subtraction, use of frequency banks, moving average filtering, and removal of corrupted signal segments have shown improvement against MA in some cases, but are not robust against motion which may have varied dynamics [19,20,21,22]. Numerous studies have investigated the use of adaptive noise cancellation (ANC) as an alternative approach to selectively filter out MA based on a specified reference signal. Reference signals used include: on-board accelerometers [3,4,5,6], a reference signal synthesized from the motion corrupted PPG signal [7,8,9,10], and a reference signal measured by an adjacent photoelectric device [11,12]. However, a separate reference signal is not always an accurate representation of the signal corruption, which can lead to unintentional filtering of a portion of the relevant PPG waveform. Alternate algorithms have been developed to extract the clean PPG waveform from the motion-corrupted signal based on fundamental components of the PPG signal. These methods include: principle component analysis (PCA) [13], independent component analysis (ICA) [14,15,16], and singular spectral analysis (SSA) [17]. Most recently, algorithms based on filtering out the motion frequency as taken from the accelerometer spectra have been useful in separating motion signal from PPG signal [23,24]. These algorithms have been proven somewhat effective during motion, but they are not currently optimized for multiple channel recordings and photoplethysmogram sensors only consist of a single pair of red (RD) and infrared (IR) light emitting diodes (LEDs) and a single photodetector (PD). When the single channel is too corrupted to reconstruct, or when the motion frequency overlaps with the PR frequency preventing motion from being filtered out, PR and SpO_2_ information may be lost, leading to dropouts during monitoring.

Multichannel devices have been used to combat motion artifacts by capturing multiple PPG waveforms simultaneously. An 8-channel PPG sensor placed on the sternum was developed that uses PCA in the frequency domain to find the most likely SpO_2_ estimation [25]. These investigators found that multichannel SpO_2_ estimates were more robust than single channel SpO_2_ estimates. A 3-channel reflectance in-ear sensor was developed and tested during standing, sitting, and walking [26]. An adaptive notch filter was implemented at the motion frequency to reduce noise contribution. These investigators found that motion-induced current was channel-specific, and that the channel with the highest power around the PR frequency varied between experimental runs. These studies showed that multichannel pulse oximetry is advantageous over single channel pulse oximetry in obtaining diverse signal information during low-motion artifact conditions.

Studies have attempted to better characterize the effects of motion artifact in pulse oximetry, and have shown that although all types of motion lead to measurement errors, a majority of errors are generated by intense, aperiodic, random movements [27]. Previously, we have shown that in a six-channel prototype reflectance-based forehead pulse oximeter, during short up-down, left-right, and circular head motion, channels responded differently to motion [28]. However, in the aforementioned work, we did not investigate whether the added complexity of a multichannel PPG approach actually produces measurable benefits compared to a single channel sensor and we also did not address the question of how to fuse the data captured by multiple channels to obtain better results than when analyzing data captured by a single channel PPG sensor. While it might seem intuitive that multiple channels are superior to a single channel, the main challenge lies in finding suitable methods to actually leverage this potential. Thus, examining how multichannel photoplethysmography responds to a wide variety of motion in comparison to the more conventional single-channel approach would help to further assess the benefit of this unique sensor design and assist in developing advanced signal extraction algorithms that make use of multichannel waveforms to improve motion tolerance in photoplethysmography including pulse oximetry.

In this paper, we explore the benefit of using a customized forehead-mounted multichannel photoplethysmographic sensor comprising six photodetectors and two pairs of red and infrared LEDs, and investigate the performance of this new wearable sensor under a variety of random motion. Since current algorithms are not necessarily able to select which measurement of PR is most correct based on signal corruption, we introduce an advanced multichannel-switching algorithm that selects the channel with the least amount of motion artifact to calculate PR every 2 s. We show that for a wide variety of random motion, channels respond differently to motion, and the multichannel estimate from the recorded sensor array outperforms single-channel estimates in terms of motion tolerance, signal quality, and PR errors.

## 2. Experimental Section

### 2.1. Device Description and Experimental Setup

#### 2.1.1. Sensor Description

**Figure 1:**
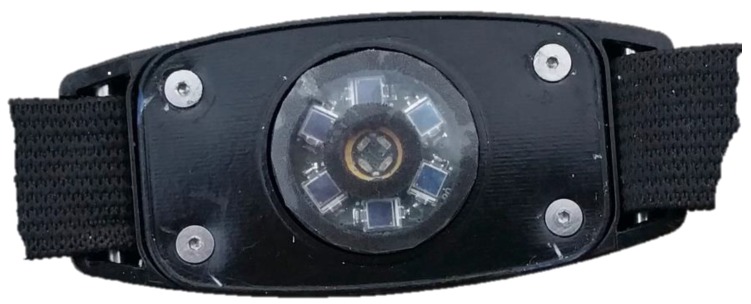
Six-photodetector (6PD) Forehead Mounted Reflectance-Mode Multichannel Photoplethysmographic (MCP) Sensor.

We have developed a customized forehead-mounted, wearable multichannel photoplethysmographic (MCP) sensor operating in reflectance mode, as shown in Figure 1. Six surface-mounted Si photodetectors (PD), each having an active area of 2.65 mm^2^, are positioned concentrically as a symmetrical array around two pairs of red (660 nm) and IR (940 nm) light emitting diodes (LEDs) at an equidistant separation distance of 10 mm [29]. Both pairs of Red and IR LEDs were turned on in pairs, at the same time, alternating between Red LEDs and IR LEDs. Two LEDs of each wavelength were used to increase total brightness. An opaque ring was incorporated to minimize direct light shunting between the LEDs and the adjacent PD array.

The sensor and battery are enclosed in a plastic casing and attached to an elastic band worn as a headband, allowing the sensor to rest comfortably on the forehead. The sensor is also equipped with an analog tri-axial MEMS accelerometer (Acc) to detect movement with respect to gravitational acceleration and to provide a reliable movement reference. When the sensor is placed on the forehead, the x-direction of the Acc corresponds to motion perpendicular to the transverse plane, the y-direction perpendicular to sagittal plane, and the z-direction perpendicular to the coronal plane. Previous data have shown that variations in sensor position and vasculature heterogeneity of the underlying tissue can cause measurement errors, as well as light diffusion by the subcutaneous tissues predominantly in the direction perpendicular to the emitting surface of the LEDs [28]. Different motions and sensor contact pressure change the optical coupling between the sensor and the underlying skin, yielding 6 independent PPG channels with different motion-corrupted waveform characteristics.

#### 2.1.2. Data Collection

Data were collected from 15 healthy volunteers between the ages of 23 and 30. Worcester Polytechnic Institute IRB approved the study protocol and informed consent was required by all subjects prior to data recording.

**Figure 2:**
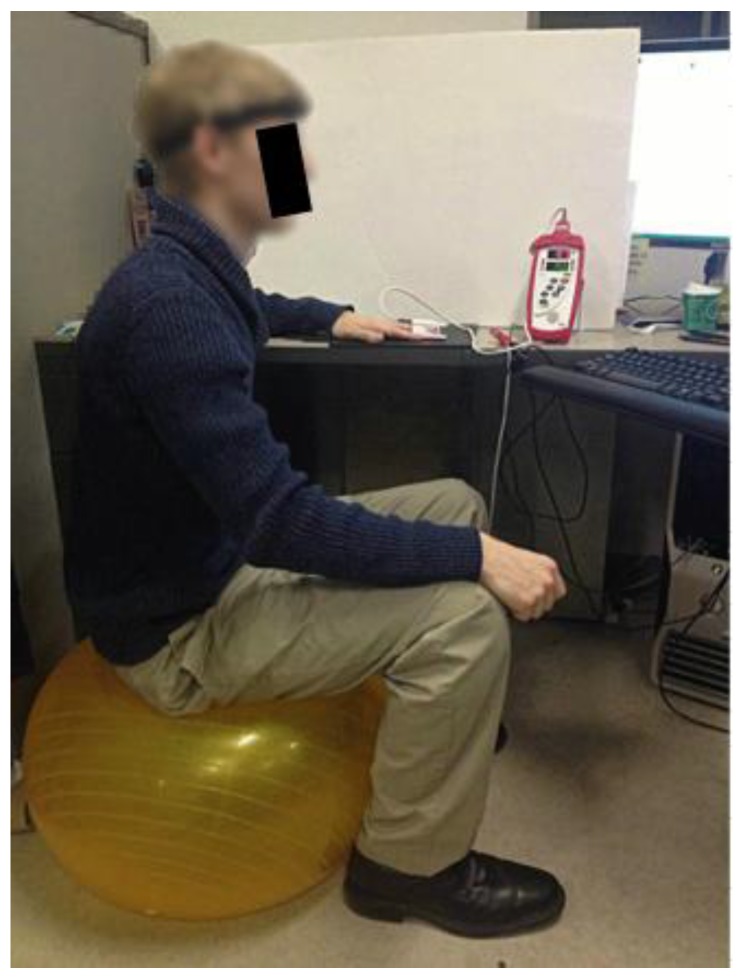
Experimental setup for generating random motion.

Subjects were asked to bounce on an exercise ball while wearing the 6PD MCP forehead sensor and a reference Masimo-57 Radical (Masimo SET^®^, Masimo Corporation, CA, USA) finger type transmittance pulse oximeter that was kept motionless by resting the left hand on a table, as shown in Figure 2. Each subject was asked to alternate between 3 min of rest and 5 min of bouncing on the exercise ball for a total of 19 min. Subjects were not instructed to move in any particular way, or to move at any given frequency, yielding data sets with different types of movement artifacts. This protocol was implemented to introduce motion artifacts with a variety of frequencies and amplitudes and an increasing and decreasing PR. Six pairs of PPG waveforms corrupted by random motion artifacts were obtained from the forehead-mounted MCP sensor. PPG waveforms from the MCP were sampled at 80 Hz, which was sufficient to recover the shape of the PPG signal. Reference PR measurements were obtained from the Masimo pulse oximeter every 2 s. All data were captured simultaneously by a PC and processed offline with MATLAB. Data from the MCP and the Masimo reference sensor were aligned by matching PR trends during rest for all data sets.

### 2.2. Methodology

The study was designed to test the response of the MCP to motion artifacts with a variety of frequencies and amplitudes. To quantify subjects’ motion, the RMS values from the on-board tri-axial accelerometer were calculated for each data set. Furthermore, because PR calculations are highly dependent on the frequency content of the IR PPG waveform, the power spectral density (PSD) was calculated for each data set, and for each IR channel, to determine how motion frequencies affect the multichannel PPG waveforms. Oxygenated hemoglobin absorbs comparably less red light than IR, so the AC component of the red PPG signal is smaller than the IR signal and the responsivity of the PD is higher in the IR region of the spectrum. Therefore, the IR PPG waveforms are used for PR measurements due to the generally better signal quality. In order to quantify instantaneous noise levels, a multichannel template-matching algorithm was developed that matches beats in a specified window to an average template representative of clean PPG morphology.

#### 2.2.1. Motion Quantification

Motion across channels was compared for all data sets using the following parameters: multichannel template-matching noise level (MCNL), RMS accelerometer amplitude, and PSD amplitude at the motion frequency.

##### Multichannel Template Matching

**Figure 3:**
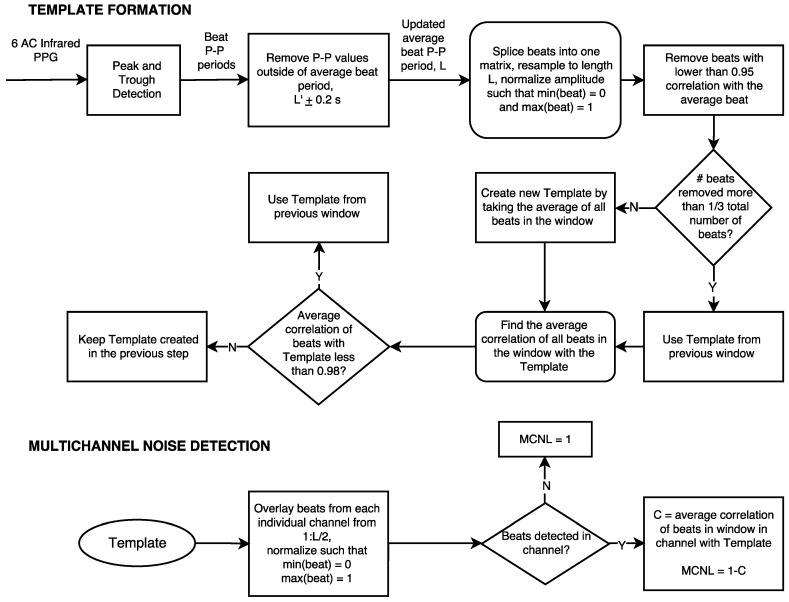
Processing of the data with the multichannel template-matching algorithm to obtain Multichannel Noise Level (MCNL) for each channel.

To quantify the noise level in each channel at a particular time point, we developed a multichannel template-matching algorithm. Algorithms have been developed based on creating a template from single channel IR PPG waveforms using an average of beats over a specified window [30]. In our algorithm, six IR PPG waveforms are used to create a template over a single time period, allowing more robust template formation. First, the raw PPG waveforms were digitally filtered with a zero-phase 6th order, 0.5 to 12 Hz Butterworth band-pass filter. The six filtered IR components served as inputs to the multichannel template-matching algorithm. Initialization of this algorithm assumes that the data starts out during rest, with clean PPG waveforms. The algorithm is divided into two separate parts, template formation and multichannel noise calculation. Both parts of the algorithm are depicted in the flowchart in Figure 3.

(a) Template Formation

Peak–rough detection was performed on each of the six IR components over a 12 s window. The peak–trough detection was implemented by alternating between finding local minima and local maxima in a ±0.25 s window. The maximum or minimum found must be the absolute maximum or minimum in this 0.5 s window preventing features, such as the dicrotic notch, to be incorrectly calculated as a peak or trough. From this, an average peak-peak period was calculated across all channels and was defined by a variable L. Peak-peak period values that are outside of L ± 0.2 s were removed to include only clean beats in the beat period calculation. The average peak-peak period is updated by taking the average of the remaining peak-peak period values. Beats in each window across all channels were segmented at the indices of each trough. Beats across all channels for the entire 12 s of data were re-sampled to be the same length (L) using a cubic spline, and normalized to have a maximum amplitude of 1 and a minimum amplitude of 0. A temporary template is created from the average of all beats in the 12 s window. The correlation between this template and all beats in the current window was computed, and if the correlation of a beat in the current window with this template was less than 0.95, the beat was removed from further calculations. If the number of beats removed were more than 1/3 of the total number of beats in the current window, the template from the previous window was used. If less than 1/3 of the beats are removed, taking the average of the remaining “good” beats in the window forms a new template. The average correlation of all individual beats in the window is again calculated with the latest template. If the average correlation across all beats in the window with this template is less than 0.98, the template from the previous window is used. If the average correlation across all beats in the window is greater than 0.98, the template from the previous step is maintained.

(b) Multichannel Noise Calculation

With the final formation of the template for a given window, the template-matching algorithm systematically overlays beats from each individual channel, cuts off each beat at L/2 in order to capture only the systolic morphology of the PPG beats, which are more indicative of clean signals, and normalizes each beat such that the minimum amplitude is 0 and the maximum amplitude is 1. If the noise is such that there are no beats detected in a given channel, the multichannel noise level (MCNL) is set to 1. Otherwise, the correlation between the template and each beat in a given channel is calculated, and the overall MCNL is calculated as (1-C), where C is the average correlation between beats in a single channel and the template.

This multichannel template-matching algorithm is implemented using a 2 s sliding window on the AC IR PPG data. Every 2 s, the channel with the lowest MCNL is selected to calculate PR. PR, in beats per minute (bpm), is calculated by dividing 60 by the average peak-to-peak period in seconds of the selected channel across the 12 s window.

##### Accelerometer Amplitude

The accelerometer signals measured during motion provide a way to quantify the amount of motion that was introduced into any particular data set. All accelerometer signals were filtered with the same filter we used to filter the PPG data, *i.e.*, a 6th order zero-phase, 0.5 to 12 Hz Butterworth band-pass filter. The RMS value of all 3 axes of the on-board accelerometer was used to quantify motion, according to Equation (1).

(1) $${\text{Accel}}_{\text{RMS}}=\sqrt{\frac{1}{3}\left({\left({\text{Accel}}_{X}\right)}^{2}+\;{\left({\text{Accel}}_{Y}\right)}^{2}+{\left({\text{Accel}}_{Z}\right)}^{2}\right)}$$

Although these calculations do not translate directly into the level of noise introduced into the corrupted PPG waveforms during motion, they help to distinguish between high motion and low motion data sets based on the overall RMS values.

##### Motion Frequency Differences

The power spectral density (PSD) of each PPG waveform represents the power of the PPG waveform at each frequency. The PSD of the AC IR PPG waveform in each channel is calculated using Welch’s periodogram. The PSD of each axis of the accelerometer was also calculated using the same method. The mean pulse rate (MPR) during motion was calculated according to the reference readings from the Masimo finger sensor during motion. The motion frequency was determined by using the accelerometer PSD, and then the amplitude of the PPG waveform PSD at the motion frequency was compared.

#### 2.2.2. Pulse Rate Performance Metrics

Pulse rate error was defined as the absolute error between the PR calculated by the multichannel device and the PR from the Masimo-57 reference sensor, according to Equation (3).

(2) $${\text{Err}}_{\text{PR}}\;[\text{bpm}]=\left|{\text{PR}}_{\text{MCP}}-{\text{PR}}_{\text{Masimo}}\right|$$

This PR error was analyzed during motion using three parameters: accuracy, precision, and performance index. The Masimo-57 pulse oximeter claims a PR error tolerance of ±5 bpm during motion [31]; thus, we used a ±5 bpm tolerance for performance index to match the Masimo specifications during motion. Performance index was defined as the percentage of the measurements that have absolute relative PR errors lower than 5 bpm during motion. The percentage in performance index corresponds to the number of low-error measurements taken during motion. Accordingly,

(3) $$\text{Performance Index }[\%]=\frac{\#\text{ measurements }({\text{Err}}_{\text{PR}}\leq 5\text{ bpm})}{\text{total }\#\text{ of measurements}}\times 100$$

Accuracy was defined as the offset that a PR measurement has in relation to the reference device. In this paper, we consider accuracy as the mean absolute PR error during motion in relation to the measurements by the Masimo-57 reference sensor. Accordingly,

(4) $$\text{Accuracy }[\text{bpm}]=\text{AVERAGE}({\text{Err}}_{\text{PR}})$$

Precision was defined as the ability to make consistent measurements, representative of the spread of measurements taken around the measured value. Hence, precision was calculated as the standard deviation of the absolute error taken in relation to the Masimo-57 reference sensor. Accordingly,

(5) $$\text{Precision }[\text{bpm}]=\text{STD}({\text{Err}}_{\text{PR}})$$

To compare the multichannel estimates against the single channel estimates for each parameter, eight one-sided Student’s *t*-tests were performed: estimates from each individual channel were compared against the multichannel-switching estimate for each parameter. The mean and median PR for each data set were compared against the multichannel-switching estimate. A confidence value of 95% (α = 0.05) was used to find t-critical values for each *t*-test.

## 3. Results

### 3.1. Time-Domain PPG Waveform Differences During Motion

**Figure 4:**
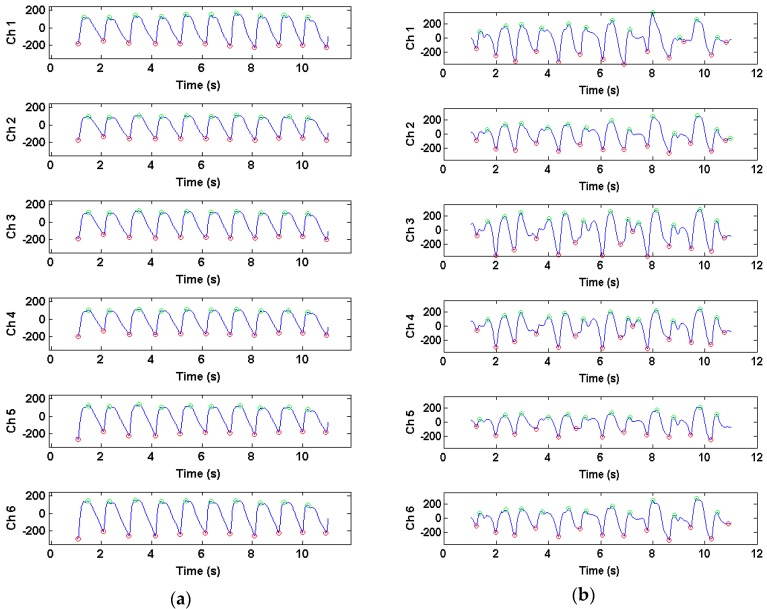
Infrared photoplethysmogram (PPG) waveform differences in all six channels recorded during rest (**a**) and motion (**b**) in a 12-s window for Data Set 10.

The infrared PPG waveforms are similar during rest across all channels, and differ in levels of signal corruption between channels during motion. Figure 4 shows infrared PPG waveforms recorded during rest and motion from Data Set 10.

### 3.2. Accelerometer Range of Motion

**Figure 5:**
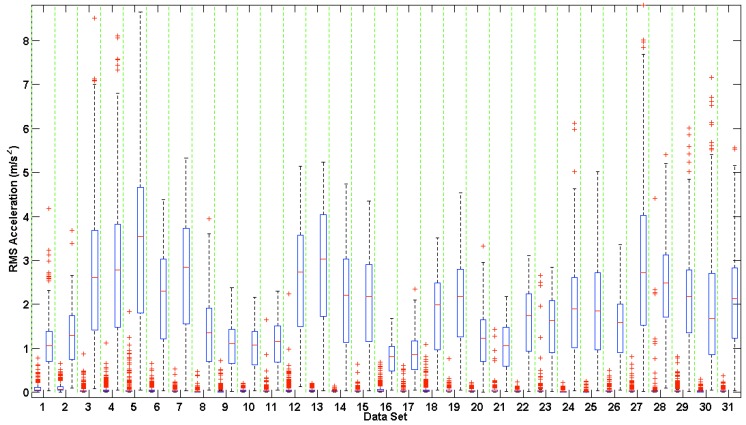
Box and Whisker plot of root mean square (RMS) accelerometer amplitudes across all data sets during rest (left side of column) and motion (right side of column). RMS values were calculated using Equation (1). The edges of the box indicate the 25th and 75th percentiles, the red line indicates the median value, and the whiskers extend to ±2.7 standard deviations. The red asterisks indicate outliers, which reside outside of the whiskers.

The RMS amplitude of the on-board tri-axial accelerometers provide an indirect measure of how much noise was introduced into the corrupted PPG waveforms in each data set, as summarized in Figure 5.

The larger the accelerometer RMS amplitude, the more intense the motion performed by the subject during testing. The accelerometer amplitude is a good indication of the level of motion introduced, but is indirectly related to the motion artifact introduced into the corrupted PPG waveform. As seen in Figure 5, about half of the data sets have a relatively small range of RMS accelerometer amplitude during motion, and median RMS accelerometer amplitudes below 2 m/s^2^. Generally, these data sets showed greater differences in motion corruption between channels. However, the pressure exerted by the headband to secure the sensor and the signal amplitude of the PPG waveform, amongst other variables, can affect the quality of the recorded PPG waveforms.

### 3.3. PPG Motion Frequency Differences

**Figure 6:**
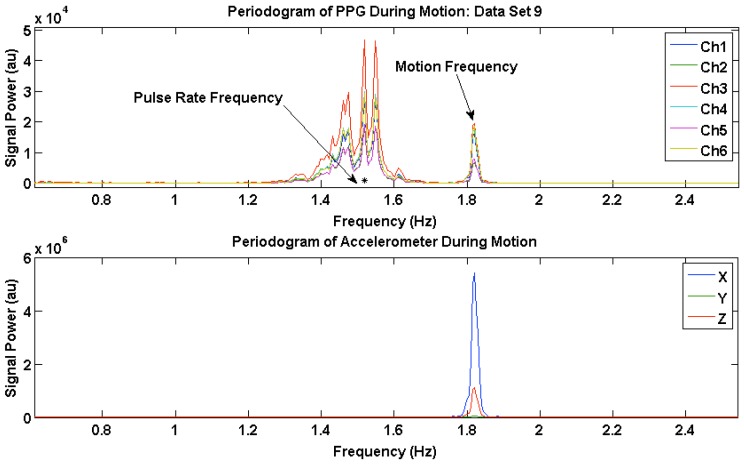
Power Spectral Density (PSD) plots of photoplethysmogram (PPG) waveforms from all six channels, and tri-axial accelerometer signals for Data Set 9.

**Figure 7:**
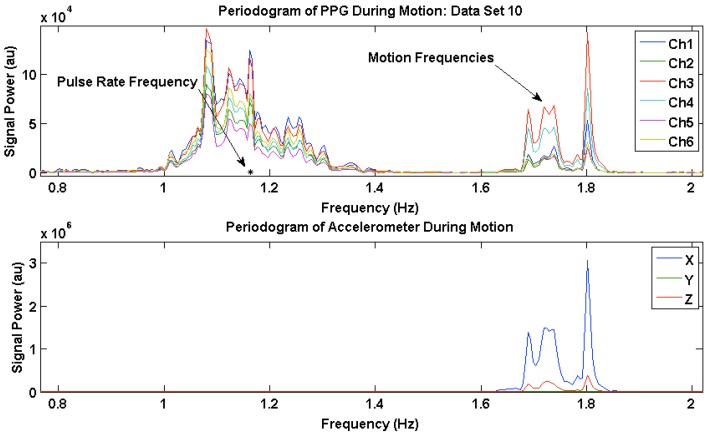
Power Spectral Density (PSD) plots of photoplethysmogram (PPG) waveforms from all six channels, and tri-axial accelerometer signals for Data Set 10.

The motion frequency differences across channels were visualized by comparing the power spectral density (PSD) of the six PPG waveforms during motion with the corresponding accelerometer signals. Figure 6 and Figure 7 show the PSD of the PPG waveforms in all six channels, and the accelerometer waveforms from two data sets, where channels have different PSD amplitudes of motion frequency. Figure 6 and Figure 7 also show the MPR frequency present in the corrupted PPG waveform, taken from the reference Masimo pulse oximeter, depicted by a black asterisk, and the motion frequency band present in the corrupted PPG waveforms across all channels as indicated by the same motion frequency power present in the Acc PSD. As shown in these figures, the majority of the motion present in our data sets occurred in the x-direction as expected, which coincides with the vertical up/down direction of the subject’s bouncing on the exercise ball.

Figure 6 shows the PR frequency and the motion frequency in the PSD during motion for Data Set 9. Channels 1 and 3 have less amplitude in the PSD at the motion frequency than the rest of the channels.

Figure 7 shows the PR frequency and the motion frequency in the PSD during motion for Data Set 10. Channels 3 and 4 have high PSD amplitude in the motion frequency, while the rest of the channels are generally lower in PSD amplitude at the motion frequency.

**Figure 8:**
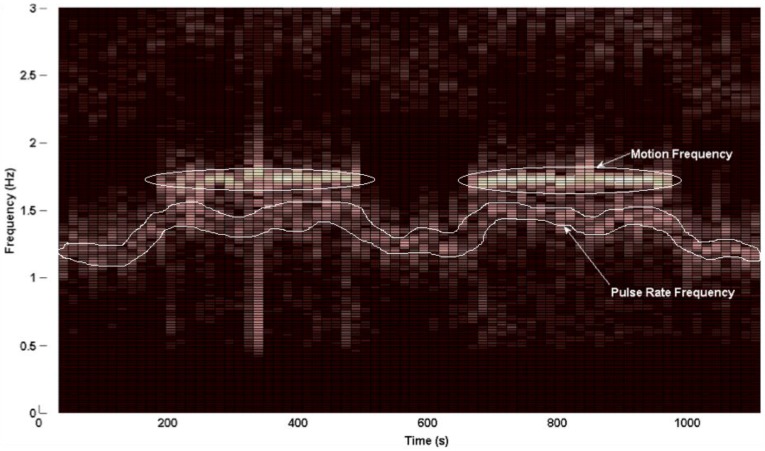
Spectrogram of photoplethysmogram (PPG) waveforms from Data Set 14 Channel 5. The dominant motion frequency appears around 1.72 Hz. Brighter coloration indicates higher power.

Figure 8 shows the entire spectrogram from Data Set 14, Channel 5. The prominent motion frequency, as seen from the Acc PSD, is 1.72 Hz.

**Figure 9:**
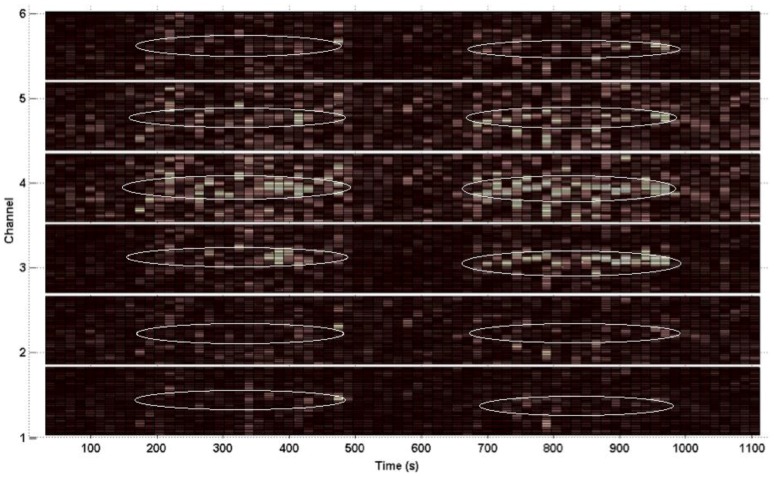
Spectrogram of photoplethysmogram (PPG) waveforms across all six channels from Data Set 14 centered around the dominant motion frequency at 1.72 Hz, circled in white. Brighter coloration indicates higher power.

Figure 9 shows the PSD centered around the dominant motion frequency for each channel. From these plots, it is evident that the motion frequency power is higher in Channels 3 and 4 and lower in Channels 1, 2, and 6.

### 3.4. Multichannel Noise Level (MCNL)

**Figure 10:**
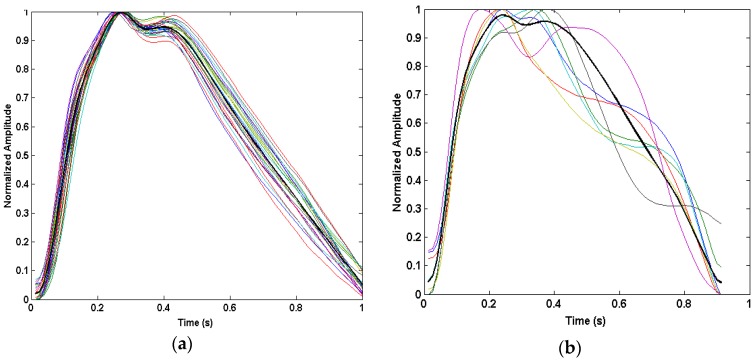
Beat detection and overlay for our Multichannel Photoplethysmogram (MCP) device during (**a**) rest and (**b**) motion.

Figure 10 shows sample PPG waveforms overlaid from all six channels during rest (left) and motion (right). During rest, the majority of beats in a window are retained to form a template from the average of the “good” beats in the 12 s window, shown in black. During motion, in this particular window, only 7 to 10 beats were kept as “good” beats, and the template from the previous window was used, as shown by the dark bold tracing.

**Figure 11:**
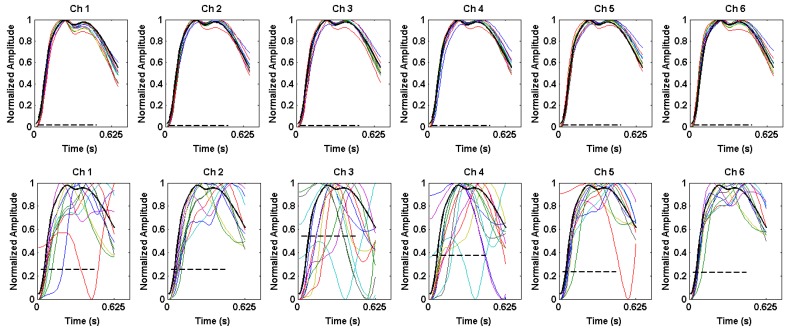
Beats in window overlaid from each individual channel during rest (**top**) and motion (**bottom**). The template used is shown by the black trace, and the Multichannel Noise Level (MCNL) for each channel is shown by the dashed black line.

During motion, some channels have higher correlations with the template than other channels, yielding different MCNL values for each channel. Figure 11 shows beats overlaid in the window separated by channel during rest (top row) and motion (bottom row). During rest, it is evident as expected that beats across all channels are highly correlated with one another and highly correlated with the template shown in Figure 11. In contrast, during motion, although some channels have beats that remain highly correlated with the template, other channels are highly corrupted by motion artifact. Therefore, the average correlation coefficient and MCNL differ between channels, allowing the algorithm to select the best possible channel for PR calculations.

**Figure 12:**
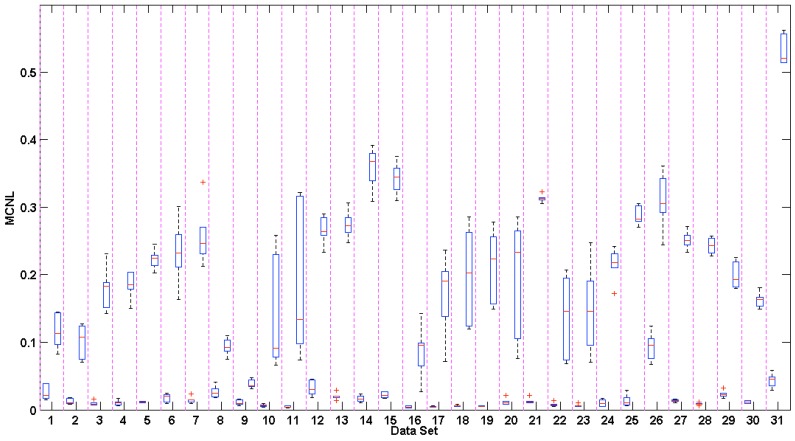
Box and Whisker plots of the average Multichannel Noise Level (MCNL) across all data sets. The average MCNL across all channels during rest and motion are shown on the left and right hand side of each column, respectively. The edges of the box indicate the 25th and 75th percentiles, the red line indicates the median value, and the whiskers extend to ±2.7 standard deviations. The red asterisks indicate outliers, which reside outside of the whiskers.

The overall MCNL across all six channels was averaged, and the corresponding Box-and-Whisker plots of the noise level during rest (left) and motion (right) are plotted in Figure 12.

**Figure 13:**
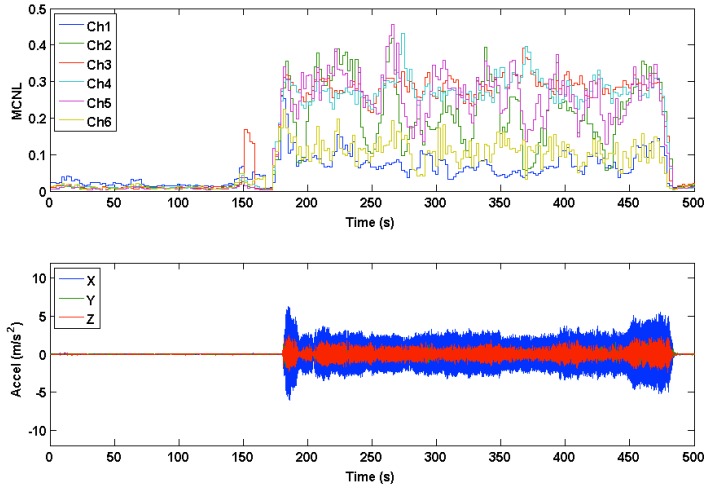
Multichannel noise level (MCNL) during rest and motion in Data Set 20. Accelerometer data are plotted below the MCNL to indicate where motion occurs.

**Figure 14:**
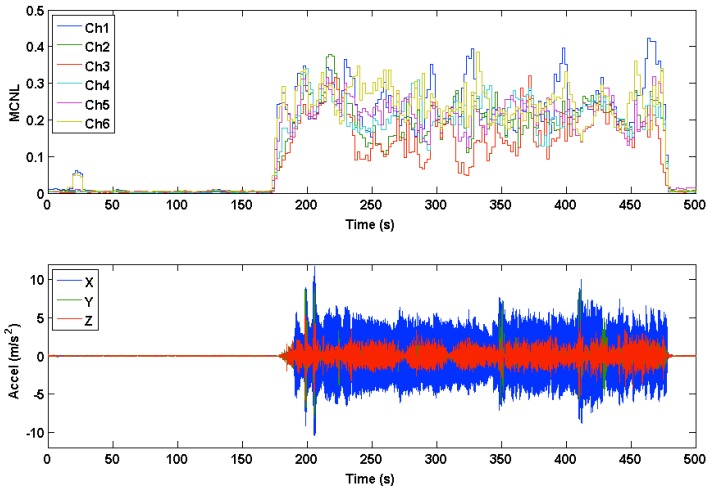
Multichannel noise level (MCNL) during rest and motion for Data Set 24. Accelerometer data are plotted below the MCNL to indicate where motion occurs.

Multichannel noise level (MCNL) plots were used to illustrate how clean or corrupted different channels were during motion based on their respective signal morphology. During clean segments of the PPG waveforms recorded during rest, beats across all channels showed a relatively high degree of correlation (C) and low MCNL values. The differences in the medians and ranges of the MCNL, calculated by the multichannel template-matching algorithm during motion, showed high variation across data sets, hence displaying the variety of motions manifested by our experimental protocol. The data sets with low MCNL values during motion tended to have low accelerometer RMS amplitudes. Figure 13 and Figure 14 show the time-series of MCNL across two different data sets, indicating low values during rest, high values during motion, and varying values across channels during motion. In Data Set 20, the channels were more consistently separated in terms of noise level, whereas in Data Set 24, the noise levels across channels were closer during motion. The filtered accelerometer signal is shown beneath the MCNL plot for motion reference.

### 3.5. PR Error During Motion

Pulse rate errors during motion were calculated in comparison to the Masimo reference sensor according to three separate parameters: performance index, accuracy, and precision. The multichannel estimate (MC) corresponds to the PR measurements taken by switching between channels every 2 s using the multichannel template-matching algorithm. Data sets 21 and 31 showed extremely low SNR, high accelerometer amplitudes during motion, high MCNL during motion, and high PR errors across all channels. Furthermore, the PPG waveforms across all channels were completely corrupted by motion artifacts; therefore, these data sets were eliminated from the statistics calculations.

#### 3.5.1. PR Performance Index (PI)

Table 1 summarizes the performance index calculated for each data set across all six channels, for the estimate of the mean PR and the median PR across all six channels, and for the multichannel-switching PR estimates. The mean and standard error of the difference between the multichannel, each individual channel, and the median and mean PR estimates are also shown. Statistical significance compared to the multichannel-switching estimate is indicated by an asterisk next to each mean PI.

We found that the performance index for the multichannel-switching estimate was on average 9.2% better than the channel with the highest average performance index and 13.6% better than the channel with the lowest average performance index. The performance index was increased by 18.9% when compared to taking the mean of PR estimates across all channels. The multichannel-switching PI was statistically significantly better than all six individual channels, the mean PR, and the median PR.

#### 3.5.2. PR Accuracy

PR accuracy was calculated for all six channels, for the mean PR, for the median PR, and for the corresponding multichannel switching estimate. Table 2 summarizes the accuracy calculated for each data set across all six channels, the estimate of the mean PR and the median PR across all six channels, and the multichannel-switching PR estimates. Statistical significance compared to the multichannel-switching estimate is indicated by an asterisk next to each mean accuracy measurement.

We found that the multichannel-switching PR estimate had the highest accuracy of PR during motion, corresponding to 1.8 bpm lower than the channel with the lowest mean error and 2.7 bpm lower than the channel with the highest mean error. In addition, we found that the multichannel-switching estimate was statistically more accurate than any individual single channel estimate, and the mean and median PR across channels for all data sets recorded during motion.

#### 3.5.3. PR Precision

PR precision was calculated for all six channels, for the mean PR, for the median PR, and for the corresponding multichannel-switching estimate. Table 3 summarizes the precision calculated for each data set across all six channels, the estimate of the mean PR and the median PR across all six channels, and the multichannel-switching PR estimates. Statistical significance compared to the multichannel-switching estimate is indicated by an asterisk next to the mean precision.

The data showed that the precision of the multichannel-switching estimate was equal to the precision of the channel with the highest precision, and better than the channel with the lowest precision by 1.7 bpm. Likewise, the multichannel estimate was statistically lower than Channel 3 in precision, but not other channels.

## 4. Discussion

Motion artifacts are the primary limiting factor in the utilization of photoplethysmography for mobile health applications. Motion artifacts are hard to quantify and filter out given the unpredictable nature of motion-induced PPG signal corruption. In this paper we obtained random, aperiodic, motion corrupted data from the forehead using a reflectance-mode, multichannel photoplethysmograph, and introduced a multichannel-switching algorithm based on previously developed template-matching algorithms. We hypothesized that the PPG waveforms in each channel during random motion would be affected differently by motion artifacts, and that the multichannel-switching algorithm would outperform single channel estimates during motion in terms of PR error and motion tolerance.

Data analysis showed that channels were different in PSD amplitude at the motion frequency. Across all data sets, depending on the severity of motion, PPG waveforms in the time domain were visually different during motion, as shown in Figure 6. Differences in amplitude at the motion frequency across channels corroborate the benefits of multichannel pulse oximetry. In particular, in the case when the motion amplitude overwhelmed a single channel, but did not affect all six channels as severely, PR measurements can still be obtained from the cleanest channel during motion. Of particular interest, when the motion frequency overlaps with the PR frequency and would be difficult to filter out if a single IR channel pulse oximeter was used, when motion amplitude did not affect all six channels to the same extent, we found that PR can still be extracted from the cleanest channel during motion with sufficient clinical accuracy.

Data sets 10, 11, 16, 17, 18, 19, 20, 22, and 23 showed significant improvement from individual channels compared to the multichannel estimates in absolute PR error—one or more individual PPG channels were above the accepted tolerance in absolute PR error, while the multichannel-switching estimate was at or below tolerance in absolute PR error. The accelerometer data shows that a wide variety of motion amplitudes were introduced across all 31 data sets. Of the nine data sets where multichannel switching decreased PR error significantly, Data Sets 18, 19, 22, and 23 had a high RMS accelerometer amplitude. Hence, accelerometer amplitude is not an accurate measure of the level of motion artifact corruption present in the PPG waveform, or an indication of how the multichannel-switching approach will affect estimated PR errors during motion.

The Box and Whisker plots of the MCNL showed a wide range of MCNL values for Data Sets 10, 11, 17–20, 22, and 23. These data sets showed significant improvement when the multichannel switching estimate was implemented. We found that the multichannel approach shows the most improvement when channels differ significantly in signal quality and morphology, resulting in a high variance of MCNL values during motion. Particularly, shown in Figure 11, when the MCNL is low for some channels during motion and high for other channels, the multichannel switching algorithm can choose automatically the channel with the least amount of motion corruption from which to calculate the most accurate PR values.

Although the benefit in performance index during motion in term of PR error differed between data sets, when all data sets are considered, the multichannel switching estimate performed better in performance index over every individual channel by at least 9%. The benefit of multichannel-switching in PR accuracy during motion also varies across data sets, but was an improvement when all data sets were considered over every individual channel by at least 1.9 bpm during motion. For both performance index and accuracy parameters, we found that the multichannel-switching estimate was statistically significantly better than all 6 individual channels, and from both the mean PR estimates and the median PR estimates. The multichannel-switching algorithm did not outperform individual channels, or the mean or median PR across channels in precision, but was not worse in comparison to any individual channel. Therefore, the precision is the same as a standard pulse oximeter, but we believe that the benefit in performance index and accuracy achieved is enough to merit the multichannel-switching approach.

The motion frequency during the majority of data sets remained relatively constant, and the majority of the motion remained in the x-direction. This led to the PPG channels with low-error PR remaining the same for the duration of motion in a number of data sets. Data sets with frequently changing motion frequency, as seen in data taken during real field motion or transportation, would implement the use of real-time channel-switching more often. And, future sensors that make use of multiple sensing sites or a number of LED-PD pairs spread out across the wrist or forehead would lead to greater differences in channels and more diverse motion tolerance, theoretically leading to further improved PR measurements during motion by implementing our multichannel-switching algorithm.

It is evident that the multichannel-switching approach presented in this paper has limitations when every channel is severely corrupted by motion artifacts. Generally, data sets with less severe motion and more significant differences in signal quality between channels will benefit more from the application of the proposed multichannel-switching algorithm during motion. Nonetheless, we think that that signal reconstruction techniques developed by our group may take advantage of the varying motion frequency content present in the six independent PPG channels to improve PR measurements during severe motion artifacts.

## 5. Conclusions

In this paper, we explored the benefit of using a forehead-mounted multichannel photoplethysmographic sensor and investigated the performance of this wearable sensor under a variety of random, aperiodic motion. In order to investigate the feasibility of the proposed MCP sensor, we introduced an advanced multichannel-switching algorithm that selects the channel with the least amount of motion artifact to calculate PR every 2 s. We showed that for a wide variety of random motion, channels had varying amounts of signal power at the motion frequency, and that the multichannel estimate outperforms single channel estimates in terms of motion tolerance and PR errors during motion. Although the overall benefit of multichannel-switching during motion differed between data sets, the multichannel-switching estimate from the recorded PPG array outperformed each individual channel, and the mean and median of PR across channels, during motion in accuracy and performance index of PR measurements. From a practical perspective, our multichannel algorithm allows potential real-time automatic selection of the best signal fidelity channel among the six channels at each time point. Without this algorithm, a post processing stage requires a user to select which channel exhibits the minimal MA at each time point, which is a time-consuming process. These data show promise in channel switching for increased motion tolerance in PR calculations, and provide a basis for future investigation of multichannel PPG-based algorithm development.

## Figures and Tables

**Table 1:** Performance index calculated for each individual channel, for the mean and median of PR measurements, and for the multichannel-switching estimation (MC) during motion.

	Ch 1	Ch 2	Ch 3	Ch 4	Ch 5	Ch 6	Mean	Median	MC
Mean (all)	57.8%	56.2%	53.3%	54.9%	56.4%	57.9%	48.8%	55.6%	66.4%
Mean (excluding 21 and 31)	* 61.7%	* 59.9%	* 56.7%	* 58.5%	* 60.1%	* 61.7%	* 52.0%	* 59.2%	70.9%
Mean diff.	9.24%	11.02%	14.15%	12.35%	10.78%	9.15%	18.85%	11.66%	
Std Err diff.	3.05%	3.39%	4.94%	4.99%	3.98%	2.53%	4.56%	3.55%	

* indicates statistical significant compared to the multichannel-switching estimate.

**Table 2:** Accuracy of PR for each individual channel, for the mean and median of PR measurements across channels, and for the multichannel-switching estimate (MC).

	Ch 1	Ch 2	Ch 3	Ch 4	Ch 5	Ch 6	Mean	Median	MC
Mean (all)	9.7	9.5	10.2	9.6	9.4	9.6	9.3	9.0	7.7
Mean (excluding 21 and 31)	* 8.1	* 8.0	* 8.8	* 8.1	* 7.9	* 8.1	* 7.8	* 7.5	6.1
Mean diff.	2.0	1.9	2.7	2.0	1.8	2.0	1.7	1.3	
Std Err diff.	1.0	0.7	1.1	1.1	0.6	0.9	0.5	0.5	

* indicates statistical significant compared to the multichannel-switching estimate.

**Table 3:** Precision of PR for each individual channel, for the mean and median of PR measurements across channels, and for the multichannel-switching estimate (MC).

	Ch 1	Ch 2	Ch 3	Ch 4	Ch 5	Ch 6	Mean	Median	MC
Mean (all)	5.7	6.4	7.3	6.6	6.5	5.7	5.4	5.8	5.7
Mean (excluding 21 and 31)	5.6	6.3	* 7.3	6.6	6.5	5.7	5.3	5.8	5.6
Mean diff.	0.0	0.7	1.7	0.9	0.9	0.1	−0.3	0.2	
Std Err diff.	0.4	0.5	0.8	0.8	0.5	0.4	0.5	0.5	

* indicates statistical significant compared to the multichannel-switching estimate.

## Abbreviations

The following abbreviations are used in this manuscript:

SpO_2_	Arterial Blood Oxygen Saturation
PR	Pulse rate
PPG	Photoplethysmogram
PD	Photodetector
LED	Light emitting diode
Acc	Accelerometer
MA	Motion Artifact
ANC	Adaptive Noise Cancellation
PCA	Principle Component Analysis
ICA	Independent Component Analysis
SSA	Singular Spectral Analysis
RMS	Root Mean Square
SNR	Signal-to-Noise Ratio
MPR	Mean Pulse rate
PSD	Power Spectral Density
MCNL	Multichannel Noise Level
IR	Infrared
RD	Red
PI	Performance Index
MC	Multichannel Estimate
MCP	Multichannel Photoplethysmogram
